# Targeting the Menin–KMT2A interaction in leukemia: Lessons learned and future directions

**DOI:** 10.1002/ijc.35332

**Published:** 2025-01-30

**Authors:** Florian Perner, Jayant Y. Gadrey, Scott A. Armstrong, Michael W. M. Kühn

**Affiliations:** ^1^ Hematology, Hemostasis, Oncology and Stem Cell Transplantation, Hannover Medical School (MHH) Hannover Germany; ^2^ DGHO, Deutsche Gesellschaft für Hämatologie und Medizinische Onkologie e.V. working group, Clinical and Translational Epigenetics Berlin Germany; ^3^ Department of Medicine Tufts Medical Center Boston Massachusetts USA; ^4^ Department of Pediatric Oncology, Dana‐Farber Cancer Institute, Division of Hematology/Oncology Boston Children's Hospital and Harvard Medical School Boston Massachusetts USA; ^5^ Department of Hematology and Medical Oncology University Medical Center, Johannes Gutenberg‐University Mainz Germany

**Keywords:** ALL, AML, Bleximenib, KMT2A, leukemia, Menin, MLL1, NPM1, NUP98, resistance, Revumenib, Ziftomenib

## Abstract

Chromosomal rearrangements involving the Mixed Lineage Leukemia gene (*MLL1*, *KMT2A*) are defining a genetically distinct subset in about 10% of human acute leukemias. Translocations involving the *KMT2A*‐locus at chromosome 11q23 are resulting in the formation of a chimeric oncogene, where the N‐terminal part of *KMT2A* is fused to a variety of translocation partners. The most frequently found fusion partners of *KMT2A* in acute leukemia are the C‐terminal parts of AFF1, MLLT3, MLLT1 and MLLT10. Unfortunately, the presence of an KMT2A‐rearrangements is associated with adverse outcomes in leukemia patients. Moreover, non‐rearranged KMT2A‐complexes have been demonstrated to be crucial for disease development and maintenance in NPM1‐mutated and NUP98‐rearranged leukemia, expanding the spectrum of genetic disease subtypes that are dependent on *KMT2A*. Recent advances in the development of targeted therapy strategies to disrupt the function of KMT2A‐complexes in leukemia have led to the establishment of Menin–KMT2A interaction inhibitors that effectively eradicate leukemia in preclinical model systems and show favorable tolerability and significant efficacy in early‐phase clinical trials. Indeed, one Menin inhibitor, Revumenib, was recently approved for the treatment of patients with relapsed or refractory KMT2A‐rearranged acute leukemia. However, single agent therapy can lead to resistance. In this Review article we summarize our current understanding about the biology of pathogenic KMT2A‐complex function in cancer, specifically leukemia, and give a systematic overview of lessons learned from recent clinical and preclinical studies using Menin inhibitors.

## Graphical abstract

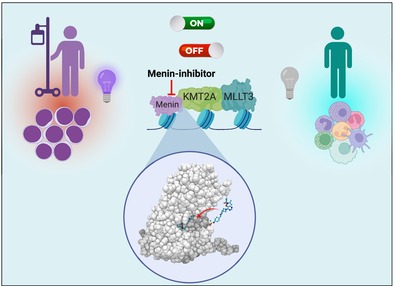

## 1 ONCOGENIC KMT2A‐COMPLEXES IN LEUKEMIA

### 1.1 *KMT2A* ‐rearrangements in leukemia development

In mammalian cells six members of the Mixed‐Lineage Leukemia (MLL)‐family (MLL1/*KMT2A*, MLL2/*KMT2B*, MLL3/*KMT2C*, MLL4/*KMT2D*, SETD1A/*KMT2F* and SETD1B/*KMT2G*) encode for methyltransferases that catalyze Histone 3 lysine 4 mono‐, di‐ and tri‐methylation (H3K4me^1/2/3^) at promotors and enhancer regions and influence transcription. Several members of this gene family have been implicated to play a role in developmental disorders and in the emergence and maintenance of cancer.^1^, ^2^, ^3^ *KMT2A* is of particular interest since it was found in the early 1990s to be part of recurrent fusion oncogenes that occur in acute myeloid‐ and lymphoid leukemia (AML/ALL).^4^, ^5^, ^6^, ^7^ KMT2A is encoded by a locus on chromosome 11q23 and the protein catalyzes H3K4me^3^ at promotor regions of active genes.^8^ Of note, KMT2A function is dispensable for proper transcription activation at the majority of protein coding genes (95%) yet is crucial for gene expression of a small set of developmentally important genes, including *HOX*‐genes.^9^ Consequently, conventional knockout mouse models of *Kmt2a* are embryonic lethal due to fatal defects in primitive hematopoiesis.^10^, ^11^, ^12^ Conditional inactivation of *Kmt2a* in adult mice allowed grossly normal hematopoiesis in the bone marrow but self‐renewal capacity of *Kmt2a* ^‐/−^ adult stem cells was impaired as they were unable to compete against WT cells in transplantation assays.^13^

**FIGURE 1:**
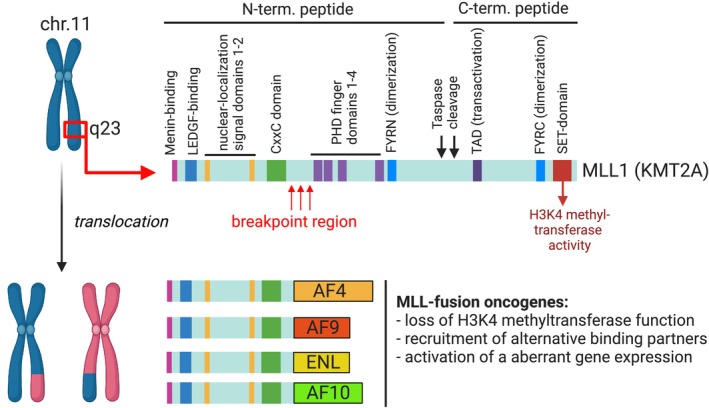
The *KMT2A* gene and development of KMT2A‐rearrangements. Schematic depiction of the gene product of KMT2A1 (*KMT2A*) with annotation of different functionally important domains (top panel). Chromosomal breaks and subsequent translocation leading to the formation of an KMT2A‐fusion oncogene by rearrangement of AFF1, MLLT3, MLLT1 or MLLT10 (most frequent fusion partners) to the N‐terminal portion of KMT2A (bottom panel).

**FIGURE 2:**
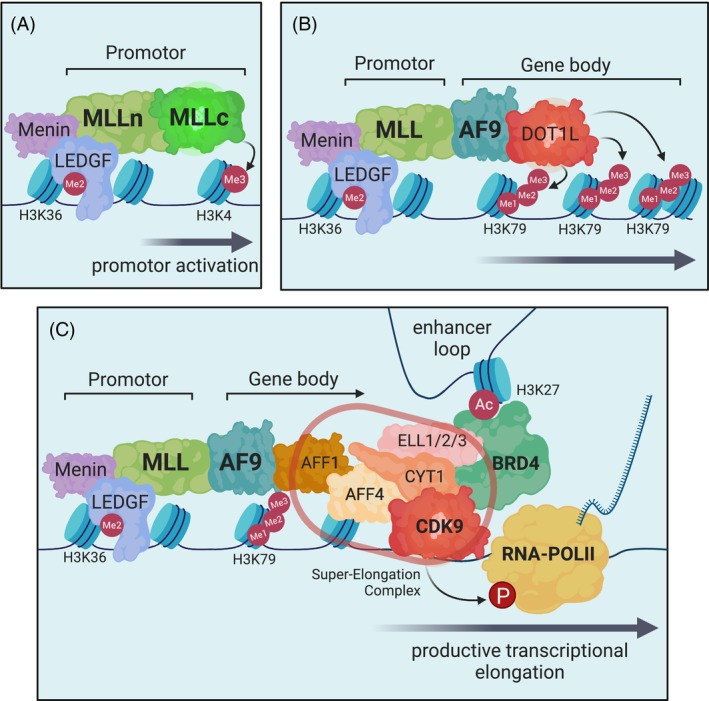
Assembly and function of normal and rearranged KMT2A‐complexes. (A) Schematic of the WT‐KMT2A heterodimer of the N‐terminal (MLLn) and C‐terminal (MLLc) KMT2A cleavage fragment catalyzing H3K4me3. (B) Schematic of the KMT2A::MLLT3 fusion in complex with DOT1L catalyzing H3K79me2. (C) Schematic of the KMT2A::MLLT3 fusion recruiting the Super‐Elongation complex (SEC) to drive transcriptional elongation of target genes.

The gene product of *KMT2A* at 11q23 (KMT2A protein) is cleaved by threonine aspartase 1 (Taspase 1) to NH_3_‐ and COOH‐terminal fragments, that associate via the FY‐rich domains (FYRN and FYRC) to form a functional heterodimer^14^, ^15^, ^16^ (Figure 1). The C‐terminal KMT2A peptide is crucial to catalyze H3K4me^3^ at promotors via its SET‐domain^17^ (Figures 1 and 2A). Importantly, the endogenous methyltransferase activity of KMT2A requires association with the C‐terminal binding partners WDR5, RBBP5 and ASH2L for efficient K3K4 methylation.^18^, ^19^ The NH_3_‐terminal portion of KMT2A supports chromatin association via Menin‐ and LEDGF‐binding, as well as via the CxxC‐domain that binds non‐methylated CpG‐islands^20^, ^21^, ^22^ (Figures 1 and 2A).

Chromosomal translocations involving the 11q23 locus produce oncogenes with diverse translocation partners^3^, ^23^ (Figure 1). The consequence of these translocations are oncogenic fusion proteins that have lost H3K4‐methyltransferase function but can associate with KMT2A‐ target genes and recruit multiple chromatin‐associated protein complexes that drive aberrant gene expression.^3^, ^20^ To date, over 100 KMT2A‐fusion partners have been identified. The most frequently found fusion partners of *KMT2A* are AFF1 (36%), MLLT3 (19%), MLLT1 (13%) and MLLT10 (8%)^20^, ^24^ (Figure 1).

When these KMT2A‐fusion oncogenes are expressed in murine hematopoietic stem‐ or progenitor cells they are sufficient to induce highly aggressive acute leukemias without cooperating genetic events.^25^, ^26^, ^27^, ^28^, ^29^, ^30^, ^31^, ^32^ Gene expression profiling of *KMT2A*‐rearranged leukemias revealed an aberrant transcriptional signature including induced expression of *HoxA*‐cluster genes and their co‐factor *Meis1*.^33^ The induction of this KMT2A‐fusion driven gene expression program was capable of transforming hematopoietic stem‐ and multipotent progenitor cells as well as committed Granulocyte‐monocyte progenitors (GMPs) into leukemic stem cells.^26^, ^34^ Importantly, forced expression of *Hoxa9* in combination with the co‐factors *Meis1* or *Pbx3* alone was sufficient to induce leukemia,^35^, ^36^ establishing those genes as central functional drivers in *KMT2A*‐rearranged leukemia by shaping the leukemic enhancer landscape.^37^

*KMT2A*‐rearrangements are found in approximately 10% of all human acute leukemias (both AML and ALL), while being particularly frequent in infant (age <1 year) acute leukemias (>70%).^34^ The detection of these oncogenes is associated with adverse outcomes in patients, particularly in infants.^34^, ^38^, ^39^ This demonstrates the urgent need for a better understanding of molecular mechanisms underlying the pathogenesis of *KMT2A*‐rearranged leukemias in order to develop more effective molecularly targeted therapeutic approaches.

### 1.2 Epigenetic and transcriptional re‐programming by KMT2A‐fusion complexes

As described above, KMT2A associates with non‐methylated CpG islands, Menin and LEDGF in order to bind promotor regions and catalyze H3K4me^3^ (Figure 2A) as a prerequisite for productive transcription by RNA‐polymerase II (RNA‐PolII).^40^ The exact mechanisms by which KMT2A or KMT2A‐fusion proteins are recruited selectively to their target genes are still unclear. Nevertheless, it is evident that CpG‐island association via the CxxC domain, binding to LEDGF, which recognizes and reads H3K36me^2^ at promotor regions, and binding to the chromatin adapter protein Menin is crucial for chromatin association and induction of gene expression driven by KMT2A^2^, ^8^, ^22^, ^41^, ^42^, ^43^, ^44^ (Figure 2A).

In contrast to wildtype, KMT2A‐fusion proteins are capable of directly recruiting alternative binding partners to target genes. Of note, the vast majority of the frequently occurring fusion partners of KMT2A are members of either the DOT1L‐methyltransferase‐complex (e.g., MLLT3, MLLT10, MLLT1, MLLT6) or the Super‐Elongation‐complex (SEC) (e.g., AFF1, ELL, AFF3, AFF4).^24^ DOT1L is the only histone‐methyltransferase, that catalyzes H3K79me^1^, ‐me^2^ and ‐me^3^ (Figure 2B). H3K79me is broadly distributed over the gene body and is strongly associated with active transcription.^45^, ^46^, ^47^, ^48^ Although, H3K79me is present at a large number of highly transcribed genes (including housekeeping genes), it is not ultimately required for transcription and adult tissues, except the hematopoietic system, tolerate knockout of DOT1L.^49^ *KMT2A*‐rearranged leukemia cells on the other hand are highly addicted to DOT1L for the maintenece of oncogenic gene expression.^25^, ^27^, ^50^, ^51^, ^52^, ^53^, ^54^ Therefore, genetic inactivation or pharmacologic inhibition of DOT1L has been established as a tool to target the KMT2A‐fusion driven transcriptional program.^52^, ^55^, ^56^, ^57^ Several KMT2A‐fusion oncogenes can recruit DOT1L, for example, via MLLT3, MLLT10, MLLT1 or MLLT6^50^, ^52^, ^58^, ^59^, ^60^, ^61^, ^62^ and thereby directly drive K3K79 methylation at target genes (Figure 2B). Of note, even KMT2A‐fusions that cannot directly recruit DOT1L (e.g., *KMT2A*::*AFF1*) have been reported to form DOT1L‐containing complexes and are typically sensitive to enzymatic DOT1L‐inhibition.^25^, ^27^, ^51^, ^63^, ^64^ In addition to DOT1L recruitment, several *KMT2A*‐fusion oncogenic complexes can directly recruit the Superelongation‐complex (SEC), that activates productive transcriptional elongation at target genes via Ser2‐phosphorylation of RNAPolII by CDK9 (Figure 2C).^59^, ^63^, ^65^, ^66^, ^67^, ^68^, ^69^, ^70^ KMT2A::MLLT3 and KMT2A::MLLT1 can bind AFF1, a part of the SEC‐backbone, via the same binding site that is utilized to bind to DOT1L.^71^, ^72^ Therefore, one molecule of KMT2A::MLLT3 or KMT2A::MLLT1 can either bind to DOT1L or the SEC complex. Presumably, those alternative complexes co‐exist at genomic loci at the same time since several KMT2A‐fusion molecules/complexes may cluster at promotor regions of a given gene. In contrast, KMT2A::AFF1, ‐ELL or ‐AFF4 fusion proteins directly recruit the SEC, since the fusion partner itself is part of the complex.^64^, ^73^, ^74^

Consequently, by recruitment of DOT1L as well as the SEC via different mechanisms, KMT2A‐fusion proteins can modify chromatin architecture and directly drive transcriptional elongation at KMT2A‐target genes for the initiation and maintenance of leukemia (Figure 2B,C). Recent work in a leukemia model system in which the KMT2A::MLLT3 oncogene was expressed fused to a degron‐tag, for the first time shed light on the hierarchy of chromatin‐related versus transcription‐related events at KMT2A‐fusion bound genes.^70^ Reduction of RNA‐PolII mediated transcription was a primary event observed as early as 30 min. after small‐molecule triggered proteasomal degradation of KMT2A::MLLT3 before relevant changes in chromatin landscape could be observed.^70^ These findings highlight the explicit importance of direct transcriptional control by KMT2A‐fusion oncogenes and demonstrate that the oncogenic protein complex needs to be physically disassembled to shut down pathogenic gene expression.

### 1.3 KMT2A‐complexes as dependencies in non‐KMT2A‐rearranged malignancies

**FIGURE 3:**
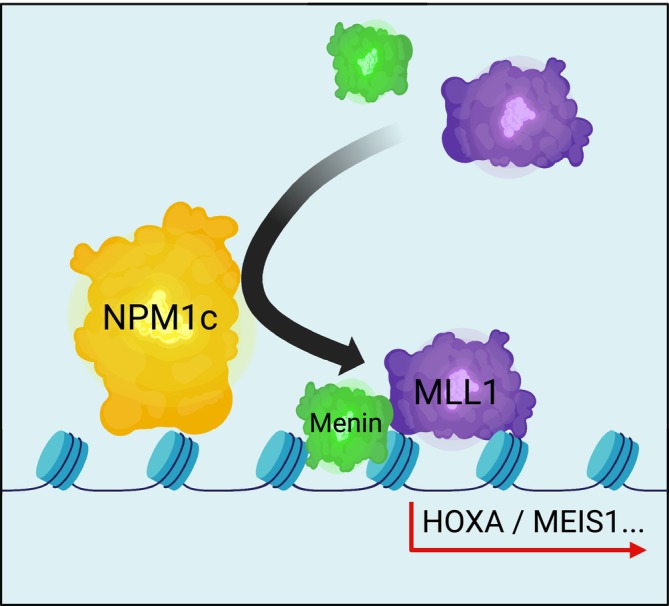
KMT2A‐complexes in NPM1‐mutated leukemia. Schematic of the assembly of WT‐KMT2A complexes recruited by mutant NPM1c in leukemia.

In contrast to KMT2A‐fusion complexes in leukemia, the relevance of WT‐KMT2A/Menin complexes remain controversial in many cancer types. The involvement of KMT2A in regulating expression of *HOX*‐ and other genes important for development and stem cell function makes it an interesting epigenetic writer in a variety of cancer types and genomic backgrounds. It has become clear, that Acute Myeloid Leukemia (AML) driven by mutations in the NPM1 gene (*NPM1c*) also express a HOX/MEIS1‐dominated gene expression program and are strongly dependent on *KMT2A*.^75^, ^76^, ^77^ In fact, many key molecular features that had been described in the context of KMT2A‐fusion proteins are highly similar to those observed in NPM1‐mutated leukemias.^75^, ^76^, ^78^ Specifically, the NPM1c oncogene drives self‐renewal of hematopoietic stem‐ and progenitor cells by inducing expression of *HOX*‐genes, *MEIS1*, *PBX3* and others via KMT2A and Menin.^76^, ^78^ Similar to KMT2A‐fusion driven disease, leukemia cell proliferation and self‐renewal as well as the underlying gene‐expression program could be abrogated by genetic inactivation of *KMT2A* or *MEN1* as well as by the pharmacologic inhibition of the KMT2A–Menin interaction.^75^, ^76^, ^78^ Interestingly, this subtype of leukemia has also been shown to be responsive to inhibition of DOT1L.^75^, ^79^, ^80^ Chromatin occupancy and rapid protein degradation experiments recently revealed that mutant NPM1 physically binds to *HOX*‐ and *MEIS1* promotors to activate its target genes^81^, ^82^ (Figure 3). Mechanistically, mutant NPM1 binds to *HOX* promotors in part via its acidic stretch domain 2 (AS2). Small‐molecule mediated proteasomal degradation of NPM1c led to a rapid breakdown in RNA‐PolII mediated transcription before chromatin state changes could be observed,^81^ analogous to the observations in *KMT2A::MLLT3* transformed cells.

Accumulating evidence further demonstrated that other drivers of leukemia also hijack WT‐Menin‐KMT2A complexes in a similar way to drive *HOX*/*MEIS1* gene expression. Leukemias with *NUP98*‐rearrangements express NUP98‐fusion proteins and are associated with a devastating prognosis particularity in children. Like NPM1c, *NUP98*‐fusion proteins and *UBTF*‐tandem duplications bind to chromatin and depend on KMT2A and Menin for target gene expression.^83^, ^84^ Of note, also these subtypes of leukemia are sensitive to genetic and pharmacologic disruption of the Menin‐KMT2A complex.

These observations clearly demonstrate that a diverse spectrum of oncoproteins belonging to unrelated families of proteins can utilize a common mechanistic principle to subvert the conserved functions and binding preferences of Menin‐KMT2A complexes. It also shows the explicit relevance of functional Menin‐KMT2A dependency in leukemia since together Menin‐KMT2A‐related mechanisms are important for approximately 50% of all AML cases. This highlights the opportunity for targeted therapeutic strategies.

**FIGURE 4:**
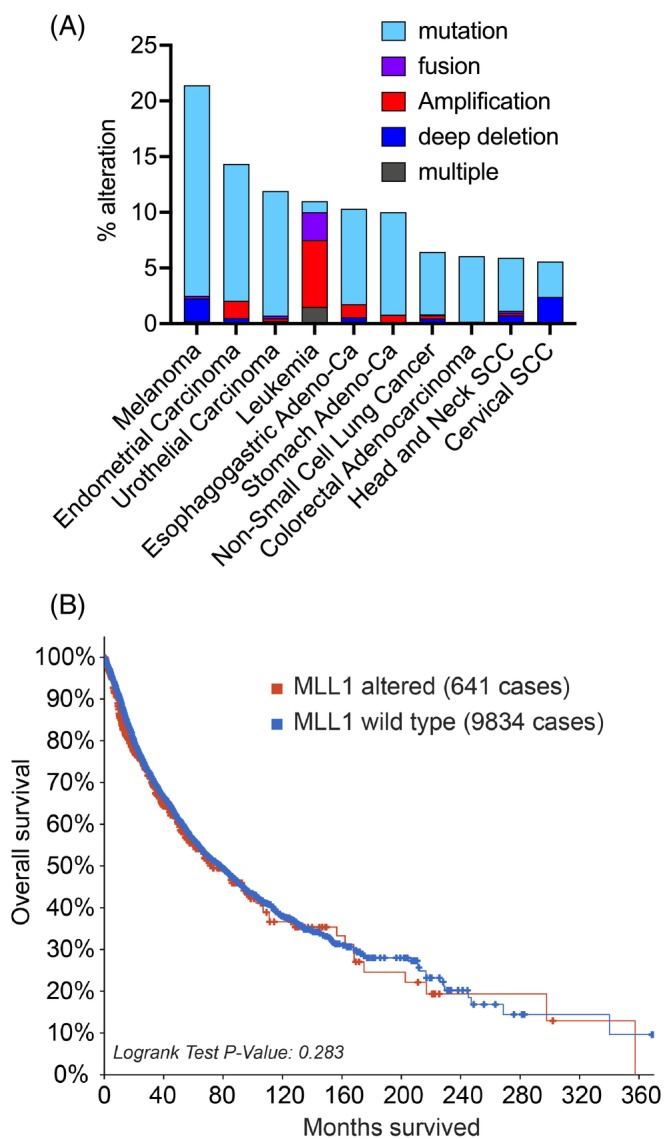
Genetic alterations of *KMT2A* in cancer. (A) Visualization of frequency and type of *KMT2A* changes in the 10 cancer types with the highest frequency of *KMT2A*‐alterations. (B) Kaplan–Meyer plot showing the survival of cancer patients with or without *KMT2A* alterations. All data has been retrieved from cbioportal.org using data from 10,475 cancer cases from the TCGA database.

**FIGURE 5:**
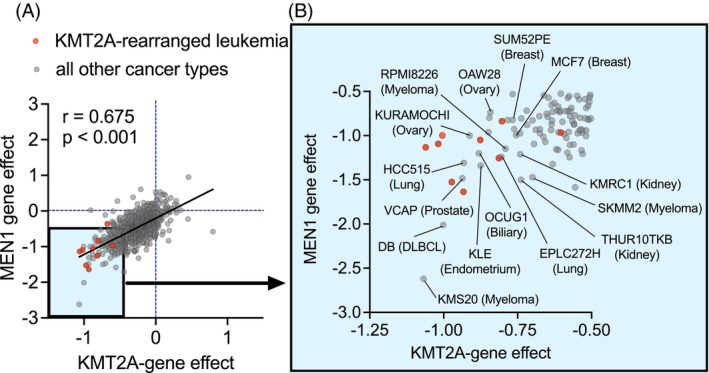
Dependency of cancer cell lines on *KMT2A* and *MEN1* (depmap.org). (A) Dot plot of dependencies of 757 cancer cell lines on *KMT2A* (x‐axis) and MEN1 (y‐axis). Plotted on the axes are the dependency score. Negative gene‐effect scores represent the degree of dependency of a given cell line. (B) Plot of cell lines that are dependent on both *KMT2A* and MEN1 (gene effect < −0.5) with annotation of the top‐dependent cell lines.

In other cancer types the relevance of KMT2A function is less clear. A number of somatic mutations in *KMT2A*‐family members affecting different domains, including the catalytic SET‐domains have been described in human cancers.^1^ The prognostic or pathophysiological relevance of the majority of these mutations which are largely heterozygous non‐ and missense mutations as well as deletions and gene‐amplifications, is not clear (TCGA database^85^;) (Figure 4). The most frequent *KMT2A* alterations in leukemia are in fact gene amplifications of *KMT2A* followed by gene fusions. Interestingly, missense or non‐sense mutations are infrequent and deep deletions are even absent in leukemia (Figure 4A), in contrast to most other cancer types. Of note, the presence or absence of *KMT2A* alterations has no impact on survival of patients across all cancer subtypes (Figure 4B). Despite the observation that *KMT2A* genomic alterations (except *KMT2A*‐rearrangements in leukemia) seem to have no overall impact on the prognosis of cancer patients, the integrity and function of the KMT2A/Menin complex seems to be important in a variety of cancer types. Querying the Cancer Dependency Map^86^, ^87^ for dependencies of 757 cancer cell lines on KMT2A and Menin revealed a clear trend of many cancer cell types to be dependent on KMT2A and/or Menin (Figure 5A). Of note, there is a strong correlation between KMT2A‐ and Menin dependency suggesting that those are indeed a reflection of addiction to the KMT2A/Menin complex rather than other potential binding partners. As expected *KMT2A*‐rearranged cells (Figure 5, red dots) are among the most dependent cell lines on KMT2A and Menin. In addition to those, a total of 123 cells lines (16.2%) exhibit a dependency on *KMT2A* (gene effect score <−0.5) and 347 cell lines (45.8%) appear dependent on *MEN1*. Among the cell lines that are dependent on both *KMT2A* and *MEN1* (103 lines, 13.6%) are cancer cell lines of Multiple Myeloma, Lymphoma, Prostate Cancer, Ovarian‐ and Endometrial carcinoma, Lung cancer, Kidney cancer, Breast cancer and others (Figure 5B). Interestingly, some of these cancer cell lines show a similar or even more pronounced genetic dependency than *KMT2A*‐rearranged leukemia lines. To date, an oncogenic role of Menin has been verified in gastrointestinal stroma tumors (GIST), breast‐ and prostate cancer.^88^, ^89^, ^90^, ^91^ Nevertheless, it is important to note that Menin's function is highly context dependent, since it is acting as an oncogene in some, but as a tumor suppressor gene in other cancer types.^92^, ^93^ Of note, Menin has been discovered as a tumor suppressor gene in a hereditary cancer predisposition syndrome “Multiple Endocrine Neoplasia” (MEN1‐syndrome) where inactivating mutations in the MEN1‐gene cause tumor development in different endocrine organs in about 80% of the gene carriers.

The mechanisms of how these tumor cells of different origin, which harbor very different oncogenic drivers, utilize the KMT2A/Menin complex to shape gene expression remains unknown. The fact that a large proportion of different cancers shows a genetic vulnerability, particularly to the loss of Menin, encourages detailed investigations of these mechanisms in non‐*KMT2A*‐rearranged cancer in the future. In the light of current advances in drug development of Menin‐inhibitors those efforts might be a fruitful approach to translate insights from chromatin biology into treatment strategies for a broader variety of malignant diseases.

## 2 MENIN INHIBITORS—TARGETED THERAPEUTICS TO DISRUPT ONCOGENIC KMT2A COMPLEXES

### 2.1 The concept of Menin‐inhibition

Menin has been identified as a crucial adapter protein that links KMT2A to chromatin.^22^, ^42^, ^94^ Therefore, the concept of targeting this adapter protein for therapeutic use has evolved more than 10 years ago. Early proof‐of‐principle studies demonstrated, that the development of molecules that block the binding pocket of Menin, which the adapter protein uses to associate with KMT2A is feasible and allows a selective disruption of oncogenic gene expression programs.^95^ In the following years these molecules were further developed towards orally bioavailable preclinical candidate molecules based on different, structurally distinct backbones.^95^, ^96^, ^97^, ^98^, ^99^, ^100^, ^101^ To date, six different Menin inhibitors entered clinical trials (Table 1).

**TABLE 1:** Overview of ongoing clinical trials using Menin inhibitors.

Serial number	NCT number	Interventions	Phases	Enrollment	Sponsor	Disease	Setting
Revumenib (SNDX‐5613)							
1	NCT06575296 (not yet recruiting)	SNDX‐5613	I	27	IIT: City of Hope Medical Center	*KMT2A*‐r or *NPM1*‐mut AML	Post‐allogeneic HSCT (maintenance)
2	NCT06229912	SNDX‐5613	II	15	IIT: MD Anderson Cancer Center	*HOX* gene upregulated acute leukemia	Relapsed/refractory
3	NCT06313437 (not yet recruiting)	SNDX‐5613, Midostaurin + Cytarabine + Daunorubicin	I	22	IIT: Dana‐Farber Cancer Institute	*NPM1‐*mut and *FLT3‐*mut AML	Newly diagnosed
4	NCT05886049	SNDX‐5613, Cytarabine + Daunorubicin	I	28	IIT: National Cancer Institute	*NPM1*‐mut andFLT3‐wt or *KMT2A*‐r AML	Newly diagnosed
5	NCT06222580	SNDX‐5613, Gilteritinib	I	30	IIT: Ohio State University Comprehensive Cancer Center	*KMT2A*‐r or *NPM1*‐mut and*FLT3*‐mutt AML	Relapsed/refractory
6	NCT06177067	SNDX‐5613, Venetoclax +Azacitidine + intrathecal chemotherapy (Cytarabine + Methotrexate)	I	24	IIT: St. Jude Children's Research Hospital	AML: *KMT2A*‐r, *NUP98*‐r, or *NPM1*‐mut AML with the following translocations: ‐*PICALM::MLLT10*, ‐*DEK::NUP214*, ‐*UBTF‐TD*, ‐*KAT6A::CREBBP*, ‐or *SET::NUP214*	Relapsed/refractory
7	NCT06226571	SNDX‐5613 (cytarabine + Daunorubicin or Idarubicin; followed by HiDAC consolidation)	I	76	Syndax Pharmaceuticals	*KMT2A*‐r, *NPM1*‐mut, or *NUP98*‐mut AML	Previously untreated
8	NCT06284486	SNDX‐5613, Venetoclax	II	8	IIT: M.D. Anderson Cancer Center	NPM1‐mut, KMT2A‐ or NUP98‐r AML	MRD positive in first or second remission
9	NCT05360160	SNDX‐5613, Venetoclax + ASTX727	I & II	43	IIT: M.D. Anderson Cancer Center	AML or MPAL	Newly diagnosed or relapsed/refractory & not eligible for high intensity chemotherapy
10	NCT05761171	SNDX‐5613, Calaspargase + Cytarabine + Fludarabine + Vincristine + Prednisolone/Prednisone + intrathecal: Methotrexate	II	78	IIT: Children's Oncology Group	*KMT2A*‐r ALL, ALAL, MPAL	Relpased/refractory
11	NCT03013998	SNDX‐5613, Azacitidine Venetoclax	I & II	N/A	IIT: Beat AML, LLC	*KMT2A*‐r or *NPM1*‐mut AML	Newly diagnosed, untreated
12	NCT05731947	SNDX‐5613, Trifluridine +Tipiracil, regorafenib	I & II	158	Syndax Pharmaceuticals	metastatic CRC or other solid tumors	Relapsed/refractory, locally recurrent or metastatic
13	NCT04065399	SNDX‐5613	I & II	413	Syndax Pharmaceuticals	*KMT2A‐*r or *NPM1*‐mut acute leukemias	Relapsed/refractory
14	NCT05918913	SNDX‐5613	Expanded Access				
Ziftomenib (KO‐539)							
15	NCT06440135	KO‐539	I	22	IIT: Massachusetts General Hospital	*KMT2A*‐r or *NPM1*‐mut AML	Post‐Allogeneic HSCT maintenance stage
16	NCT06001788	KO‐539 Fludarabine + Idarubicin + Cytarabine or LDAC or Gilteritinib	I	171	Kura Oncology, Inc.	*KMT2A*‐r or *NPM1*‐mut (±*FLT3*‐mut) AML	Relapsed/refractory
17	NCT04067336	KO‐539	I & II	199	Kura Oncology, Inc.	Phase I: AML Phase II: *NPM1*‐mut AML	Relapsed/refractory
Bleximenib (JNJ‐75276617)							
18	NCT05453903	JNJ‐75276617 (Venetoclax + Azacitidine or Cytarabine +Daunorubicin)	Ib	150	Janssen Research & Development, LLC	*KMT2A*‐r or *NPM1*‐mut AML	De novo, secondary AML, and relapsed/refractory
19	NCT04811560	JNJ‐75276617	I & II	350	Janssen Research & Development, LLC	Acute leukemia	Relapsed/refractory
BMF‐219							
20	NCT05153330	BMF‐219	I	177	Biomea Fusion	KMT2A‐r or NPM1‐mut ycute leukemia; DLBCL, MM, CLL/SLL	Relapsed/refractory
DSP‐5336							
21	NCT04988555	DSP‐5336	I & II	70	Sumimoto Pharma America, Inc.	AML, ALL, Acute leukemia of ambiguous lineage	Relapsed/refractory
BN‐104							
1	NCT06052813	BN‐104	I & II	90	BioNova Pharmaceuticals	AML, ALL, Acute leukemia of ambiguous lineage (except APML); phase II: only *KMT2A*‐r or *NPM1*‐mut acute leukemia	Relapsed/refractory

Abbreviations: ALAL, ambiguous lineage acute leukaemia; ALL, acute lymphoblastic leukemia; AML, acute myeloid leukemia; APML, acute promyelocytic leukemia; CLL, chronic lymphoid leukemia; CRC, colorectal cancer; DLBCL, diffuse large B‐cell lymphoma; FLT3‐mut., mutated FLT3‐gene; HSCT, hematopoietic stem cell transplantation; KMT2A‐r, rearranged KMT2A gene; MM, multiple myeloma; MPAL, mixed phenotype acute leukemia; NPM1‐mut., mutated Nucleophosmin gene; NUP98‐r, rearranged NUP98 gene; SLL, small lymphocyte lymphoma.

**FIGURE 6:**
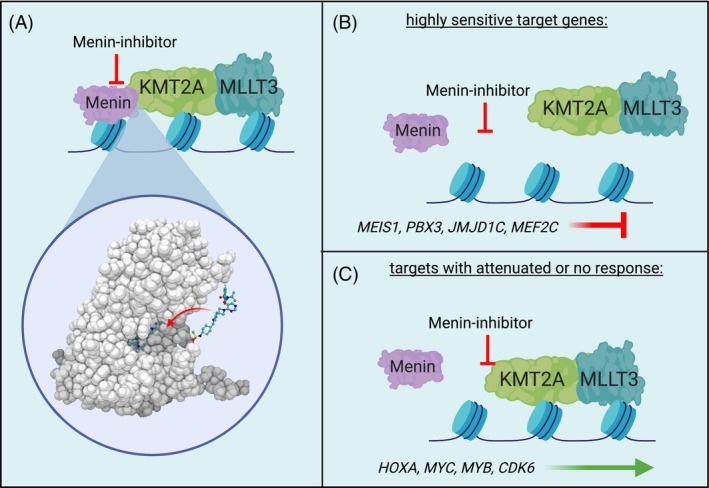
Molecular mechanisms of Menin inhibition. (A) Scheme of the Menin‐Inhibitor VTP‐50469 (analogue to Revumenib) binding to the KMT2A‐binding pocket in Menin. Structure: PDB: 6PKC (from Krivtsov et al. 2019). (B) Schematic of Menin‐KMT2A‐complex changes after Menin inhibition at highly sensitive target genes. (C) Schematic of Menin‐KMT2A‐complex changes after Menin inhibition at genes with an attenuated or no response to Menin inhibition.

These inhibitors block the physical interaction between Menin and KMT2A, thereby disassembling the protein complex on chromatin and shutting down KMT2A‐driven gene expression (Figure 6A).

An intriguing question is why Menin‐inhibition does not inflict severe toxicity in the hematopoietic system, given that KMT2A is essential for HSC self‐renewal. Recent preclinical and molecular studies have demonstrated that the gene expression changes caused by Menin inhibition are indeed highly selective and only affect a small number of target genes at early timepoints^70^, ^76^, ^78^, ^81^, ^98^ (Figure 6B, C). The molecular basis for this phenomenon remains incompletely understood, but in fact the ultimate requirement of Menin for KMT2A‐complex integrity seems to be limited to a small number of target genes (including the transcription factor *MEIS1*) that are particularly essential for leukemia cells, thereby creating an impressive therapeutic index.

### 2.2 Preclinical development of Menin‐inhibitors

In 2012, the first potent and selective Menin–KMT2A interaction inhibitor, MI2‐2, was discovered, showing activity in reducing cellular proliferation and oncogenic gene expression in KMT2A‐rearranged leukemia.^95^, ^101^ This led to further development, resulting in MI‐503 and MI‐463, two highly potent, orally available compounds with significant preclinical efficacy in KMT2A‐rearranged leukemia cell‐line xenografts.^100^ In 2016, research by Kühn et al. discovered the Menin–KMT2A interaction as a dependency of NPM1‐mutated AML and MI‐503 to be highly active against preclinical models of this most common AML subtype.^75^ More recently, MI‐3454, a structurally related compound, demonstrated potent effects on proliferation and gene expression in both human leukemia cell lines and primary patient samples.^97^ Importantly, MI‐3454 produced lasting responses in patient‐derived xenografts of both KMT2A‐rearranged and NPM1‐mutated leukemia.

Another highly specific, structurally distinct Menin–KMT2A interaction inhibitor, VTP50469, has also shown nanomolar‐range activity, effectively suppressing KMT2A‐target gene expression and cell proliferation in KMT2A‐rearranged leukemia models.^98^ In a broad range of patient‐derived xenografts, VTP50469 induced long‐lasting responses and eradicated disease in several AML and ALL grafts. Moreover, in NPM1‐mutated AML models, this molecule prevented disease progression by targeting pre‐leukemic clones and was also effective in treating established leukemia.^76^ Notably, while *HOXA*‐cluster gene expression remained largely unaffected, the expression of *MEIS1* and *PBX3* was significantly and rapidly reduced by VTP50469.

ChIP‐sequencing studies have provided insights into these differential responses, revealing that treatment with VTP50469 results in a widespread loss of Menin and DOT1L from chromatin, thus destabilizing the oncogenic KMT2A complex. Crucially, at highly responsive target genes like MEIS1 and PBX3, the inhibitor also displaces KMT2A and KMT2A‐fusion proteins.^70^, ^76^, ^98^ Therefore, the capacity of Menin inhibitors to remove KMT2A from target genes appears to be a strong indicator of their effectiveness in downregulating gene expression.

In preclinical models, various Menin inhibitors have now shown remarkable effectiveness in treating KMT2A‐rearranged, NPM1‐mutated, and NUP98‐rearranged leukemia.^83^, ^96^, ^102^

### 2.3 Clinical development of Menin‐inhibitors

The above‐described auspicious preclinical data have driven rapid progress into early‐phase clinical assessment. The AUGMENT‐101 trial, initiated in 2019, assessed the oral Menin inhibitor Revumenib (SDNX‐5613; a close homologue to the preclinical VTP50469 compound) in patients with relapsed or refractory acute leukemia and was quickly amended to enroll only patients with *KMT2A*‐rearranged and *NPM1‐mutated* leukemia. At the time of phase‐I data reporting, 68 patients had been enrolled. Revumenib was generally well tolerated, with the most common treatment‐related adverse event being a reversible prolongation of the QTc interval.^102^ On average, patients had undergone four prior treatments, with 46% experiencing relapse following allogeneic stem cell transplantation. The impact of preclinical findings on guiding trial design is evident in the promising exploratory efficacy analysis of the phase‐I data, with an overall response rate (ORR, excluding partial remission) of 53% of evaluable patients and 30% achieving a complete remission (CR) or CR with partial hematologic recovery (CRh). The median duration of response for those achieving CR or CRh was 9.1 months.^102^ In the meantime, the phase‐II part from the same trial was fully recruited. Similarly, promising results were recently published for the *KMT2A*‐rearranged patient cohort^103^: Among 94 patients treated, key grade ≥3 adverse events included febrile neutropenia (37.2%), differentiation syndrome (16.0%), and QTc prolongation (13.8%). The efficacy‐evaluable cohort (*n* = 57) showed an ORR of 63.2% with a combined CR rate (CR/CRh) of 22.8%. Notably, 68.2% of patients in combined CR also achieved undetectable residual disease.^103^ Phase‐II data of the NPM1‐mutated AML patient cohort are being analyzed and expected to be reported soon. Based on these promising results in this difficult‐to‐treat patient cohort, Revumenib received breakthrough designation from the FDA and was approved for the treatment of relapsed or refractory leukemia with KMT2A‐rearrangements by the FDA in November 2024.

Promising results from a similar phase‐I/II trial (KOMET‐001) assessing the oral Menin inhibitor Ziftomenib (KO‐539, developed from MI‐3454) in 83 heavily pretreated R/R AML patients (median number of prior therapies: 3; previous allogeneic SCT: 31%) were also recently published. The most commonly reported ≥grade 3 TRAEs were anemia (24%), febrile neutropenia (22%), pneumonia (19%), and differentiation syndrome (15%). In this first study, Ziftomenib was particularly effective in patients exhibiting the *NPM1*‐mutated AML subtype, with 35% of patients treated at the recommended phase 2 dose achieving CR (with MRD negativity in 57% of those patients).^104^ In 2024, Ziftomenib received breakthrough designation from the FDA.

Exploratory efficacy data were also reported from a phase‐I dose escalation trial assessing the oral Menin inhibitor JNJ‐75276617 (Bleximinib). Of 33 *NPM1*‐mutated or *KMT2A*‐rearranged acute leukemia patients, the ORR was 46%, with a CR/CRh rate of 27%.^105^ Table 1 provides an overview of active clinical trials assessing Menin inhibitors, including those without readily available efficacy data.

### 2.4 Adaptation and resistance to Menin inhibitors

During the AUGMENT‐101 trial it became clear, that different types of resistance to Menin inhibition are a challenge in the clinical management of patients. First, almost half of the patients do not show an objective response to Menin inhibitor monotherapy at all. This up‐front resistant subset of patients provides a significant challenge since as of now, there is no biomarker to predict this phenomenon and select patients with a higher likelihood of having a clear therapeutic benefit. Understanding those mechanisms of up‐front resistance will be of critical importance and is currently a focus of different clinical and pre‐clinical studies.

Second, leukemia cells can persist for a long period of time under Menin‐inhibitor treatment eventually giving rise to acquired drug resistance.^102^, ^106^, ^107^ Of note, aside from silencing of canonical Menin‐KMT2A‐target genes (like the HOX‐co‐factors MEIS1 and PBX3 genes) a non‐canonical Menin‐inhibitor induced cell fate program needs to be activated to cause senescence, cell cycle arrest and ultimately extinction of the malignant cell population.^108^ If the activation of this cell‐fate program is blunted by genetic or epigenetic mechanisms, leukemia cells may become resistant to the Menin inhibitor despite its retained ability to silence HOXA/MEIS1 gene expression programs.^107^, ^108^, ^109^ Similarly, an undisturbed function of Polycomb‐repressive complexes (PRC), specifically PRC1.1, seems to be required for silencing of KMT2A‐target genes upon Menin‐inhibitor treatment. In KMT2A‐ as well as NUP98‐rearranged leukemia unbiased CRISPR‐Cas9 screens have revealed that loss of PRC1.1 function renders leukemia cells resistant to Menin inhibition.^108^, ^110^, ^111^, ^112^ This is of particular importance since mutations in BCOR, a member of the PRC1 complex, are present in a relevant fraction of AML patients and may be positively selected for and cause resistance to Menin inhibition.^111^

In contrast to these adaptive mechanisms, a selective set of point mutations in the Menin binding pocket arouse during the AUGMENT‐101 trial and caused Menin inhibitor resistance. Mutations in M327, T349 and G331 were detected 38% of patients that were treated for a longer period with Revumenib.^106^ The same mutations could be identified in patient‐derived xenograft (PDX) models which acquired drug resistance and mediated a significant shift in Revumenib sensitivity when introduced into cell lines using CRISPR‐Cas9.^106^ Particularly the M327 mutations broadly caused cross‐resistance to other available Menin inhibitors, highlighting this mechanism as a highly relevant escape mechanism under Menin targeted therapy.^106^, ^107^ X‐ray crystallography and biochemical binding assays revealed that the different molecule chemotypes have distinct binding pattern to the pocket and that they are differentially affected by the established MEN1 mutations.^107^ These findings suggest that, like kinase inhibitors, a rotation of molecules and the development of second‐generation compounds may be beneficial to overcome resistance in patients that have acquired a MEN1 mutation. Furthermore, it is noteworthy that the MEN1 mutations discovered cause relative resistance with an IC50 shift in sensitivity, rather than an absolute resistance to the inhibitors. Therefore, increasing the doses, particularly using highly potent chemotypes like Bleximenib, may be another way to overcome resistance caused by MEN1 mutations.^96^

### 2.5 Rational and mechanistic combination therapies with Menin inhibitors

Combining Menin inhibitors with other active agents is expected to increase response rates, decrease persistent cells, and diminish the rate of acquired resistance by (I) diversifying selective pressure and (II) increasing the potency of functional KMT2A‐complex disruption. While first clinical trials were already initiated to combine Menin inhibitors with established chemotherapy‐based treatment regimens, preclinical research has focused on combining targeted drugs with Menin inhibitors. Synergistic Menin‐inhibitor combinations under clinical investigation: The first report of synergistic combination partners for Menin inhibitors were drugs targeting FLT3.^113^ FLT3 is a class‐III receptor tyrosine kinase and activating mutations occur in about 30% of patients with AML, co‐occur particularly with NPM1‐mutations, and internal tandem duplications (ITD) in FLT3 are associated with inferior treatment outcome.^114^ The FLT3 inhibitors Gilteritinib, Midostaurin, and Quizartinib are approved for the treatment of patients with *FLT3* mutant AML in the US and in Europe.^115^ A first preclinical study by Dzama et al. demonstrated that Menin inhibition targets *FLT3* mutations transcriptionally in *NPM1‐*mutated and *KMT2A*‐rearranged leukemias and that combined Menin‐ and FLT3‐inhibition is synergistically active in these leukemia subtypes with concurrent *FLT3* mutation. Importantly, FLT3 was shown to be a direct target of the oncogenic Menin‐KMT2A‐complex and in many cellular contexts its expression is only insufficiently repressed by Menin inhibitor treatment alone.^116^ Several independent studies have described synergy between Menin‐ and FLT3‐inhibition.^113^, ^117^, ^118^

The combination of FLT3 kinase and Menin inhibitors significantly suppressed FLT3 signaling and downstream gene expression resulting in enhanced anti‐leukemic activity. The drug combination also showed greater inhibition of cell proliferation and increased apoptosis in *NPM1* mutated and *KMT2A*‐rearranged leukemia models with *FLT3* mutations. Primary AML cells from patients with NPM1^mut^/FLT3^mut^ responded more effectively to the combined treatment than to single‐agent or control treatments, while cells without these mutations were unaffected. In vivo, this combination reduced leukemia burden and improved survival, suggesting a promising therapeutic strategy for AML with NPM1 or KMT2A‐r mutations and FLT3 mutations.^113^ Early clinical studies evaluating the combination of FLT3‐ and Menin‐inhibitors were recently or are about to be initiated but have not released data yet (Table 1).

From a clinical point of view, combining Menin inhibitors with the standard‐of‐care doublet of hypomethylating agent and venetoclax is of particular interest for the treatment of elderly non‐fit AML patients. Therefore, this combination has been addressed in various preclinical studies: First, BCL2 itself is an important target gene of the KMT2A‐fusion and a critical dependency *KMT2A*‐rearranged leukemia.^116^, ^119^ Second, Azacitidine treatment mediates downregulation of MCL1, thereby increasing the sensitivity to BCL2 inhibition.^120^, ^121^ Third, different groups independently reported a cooperation between the Menin inhibitors Revumenib, Ziftomenib or Bleximenib and BCL‐2 inhibitor Venetoclax in preclinical models of AML.^96^, ^117^, ^122^, ^123^ These studies could experimentally demonstrate synergy between Menin and BCL2 inhibition, yet the survival advantage of xenografts under this doublet treatment was modest as compared to Menin‐inhibitor monotherapy and all mice eventually succumbed to their disease.^96^, ^121^, ^122^

Introducing the synergistic triplet combination of Menin inhibitors with HMA and venetoclax (HMA / VEN) into the first‐line treatment *NPM1*‐mutated or *KMT2A*‐rearranged AML patients unfit for intensive chemotherapy is the most obvious next step in the clinical development of these agents. Preliminary data from three phase‐I clinical trials assessing the triplet combination were recently presented, all demonstrating the feasibility and promising exploratory efficacy:

The SAVE‐trial evaluated an all‐oral combination of Revumenib with Decitabine/Cedazuridine (ASTX727) and Venetoclax in fit patients with relapsed or refractory AML.^124^ Eight patients were treated across two dose levels, with no deaths from treatment‐related adverse events and a manageable safety profile, though febrile neutropenia was common. Seven out of eight patients showed a response, achieving morphologic remission, with a 100% overall response rate. Three patients moved on to stem cell transplant and measurable residual disease was undetectable in 43% of evaluable patients.^124^ Another phase Ib trial evaluated Bleximenib in combination with Azacitidine (AZA) and VEN in relapsed/refractory *KMT2A*‐rearranged or *NPM1*‐mutated AML patients.^125^ Patients underwent a median of 2 prior lines of therapy and 32% had a prior allogeneic SCT The majority of the grade 3 side effects comprised of cytopenias including febrile neutropenias (37%). Among the 34 evaluable patients for the efficacy analysis, the ORR was 79% and the CR/CRh rate was 24%.^125^

Under the umbrella of the BEAT‐AML master trial the triplet combination was moved into first‐line treatment of elderly non‐fit *NPM1*‐mutated or *KMT2A*‐rearranged patients: In this ongoing phase I trial, Revumenib is assessed in combination with Azacitidine (AZA)/VEN in patients previously untreated *NPM1‐mutated* or *KMT2A*‐rearranged leukemias.^126^ The combination was generally well tolerated; in 26 evaluable patients, the most common grade 3 or higher TRAEs were QTc prolongation (*n* = 3), febrile neutropenia (*n* = 2), GI toxicity, and differentiation syndrome (each *n* = 1). At the time of reporting, the ORR was 100%, with 96% achieving a composite CR (CRc).^126^

These promising results from Menin inhibitors in triplet combinations warrant the further exploration of these regimens in more advanced clinical trial phases aiming at getting these drugs approved. An international, multicenter, randomized phase‐3 trial by an international consortium of academic clinical trials groups is currently in preparation to evaluate the backbone of AZA / VEN with Revumenib or placebo (EVOLVE‐2). Similar trials assessing other Menin inhibitor in triplet combinations are underway.

Synergistic Menin‐inhibitor combinations for future clinical investigation: the activation of a non‐canonical KMT2A‐program is critical for the induction of senescence and cell cycle arrest under Menin inhibitor treatment.^108^ Part of this program is endogenous Cyclin‐dependent kinase (CDK) inhibitors, like CDKN2C. Failure to potently induce this program can therefore be overcome by co‐treatment with a CDK4/6 inhibitor. The CDK4/6 inhibitors Palbociclib and Abemaciclib have shown synergy with Menin inhibitors in *KMT2A*‐ und NUP98‐rearranged as well as NPM1‐mutated AML.^108^, ^117^, ^118^ Of note, CDK6 is also a key target of the Menin‐KMT2A complex,^70^, ^127^ highlighting its direct involvement in the oncogenic gene expression program.

Another class of clinically available molecules that show synergy with Menin inhibitors in *KMT2A*‐rearranged and NPM1 mutant leukemia are immunomodulatory drugs (IMIDs), like Lenalidomide, Pomalidomide or Mezigdomide.^116^, ^128^ These compounds degrade different critical zinc‐finger transcription factors. The genetic and pharmacologic dissection of this observation revealed that Ikaros (*IKZF1*) is the transcription factor relevant for the synergy with Menin inhibition. Combined Menin inhibitor and potent Ikaros degrader treatment strongly increased toxicity in leukemia cells and allowed for curative treatment of PDX, including a PDX model which had been demonstrated to succumb from *MEN1*‐mutant resistant leukemia after a relatively short latency.^128^

The enzymatic inhibition of binding partners of oncogenic KMT2A‐complexes have also shown to increase sensitivity to Menin inhibition. Inhibition of either the histone methyltransferase DOT1L or the acetyltransferase KAT6A, both co‐occupy KMT2A‐bound genes, cooperated with Menin inhibition, and increased the potency and depth of KMT2A‐target repression.^70^, ^109^, ^127^ Other combinations with epigenetic inhibitors, including BET‐inhibitors or Inhibitors of the SWI/SNF chromatin remodeling complex have also been investigated and demonstrated synergy.^127^, ^129^

## 3 CONCLUSION AND OUTLOOK

Menin inhibitors represent novel drugs with astonishing clinical activity against *NPM1*‐mutated or *KMT2A*‐rearranged leukemia in patients suffering from relapsed or refractory disease. While clinical investigation is ongoing, and these drugs will have to stand the test of randomized trials, the path of discovery may already be considered a success story: The development of these inhibitors and their introduction into clinical testing were a direct consequence of fundamental research efforts that discovered the Menin–KMT2A interaction as a dependency and therapeutic opportunity in these leukemia subtypes. Sophisticated medicinal chemistry approaches subsequently allowed the synthesis of this new class of protein–protein interaction inhibitor drugs aimed at disrupting a chromatin complex. So far, the development of Menin inhibitors has followed a textbook path that may serve as a role model for future drug developments in oncology: Unlike many other examples in modern oncology, Menin inhibitors entered clinical phase‐I trials after a very detailed preclinical characterization that concisely defined sensitive AML subtypes as well as synergistic drug combination partners. These data were building the basis for a rationale and, so far, successful design of clinical trials assessing these drugs as monotherapy or in combination. Menin inhibitors represent the first targeted drug for treating the most common *NPM1*‐mutated AML subtype.

## AUTHOR CONTRIBUTIONS

**Florian Perner:** Conceptualization; writing – original draft; writing – review and editing. **Jayant Y. Gadrey:** Data curation; writing – review and editing. **Scott A. Armstrong:** Writing – review and editing; supervision. **Michael W. M. Kühn:** Conceptualization; writing – original draft; writing – review and editing.

## FUNDING INFORMATION

This study was supported by grants from the Deutsche Forschungsgemeinschaft (DFG) to Michael W. M. Kühn: KU‐2688/2‐1 and KU‐2688/2‐2 and SFB1292/2/TP12. Florian Perner is supported by the Emmy‐Noether Programme of the German Research Foundation (DFG, PE 3217/2‐1, PN: 528168324), the DFG Research‐Unit TARGET‐MPN (PE 3217/4‐1, PN: 517204983) and the “Else Kröner‐Fresenius‐Stiftung” (2021‐EKEA.111). Scott A. Armstrong is supported by NIH grants P01 CA066996 and R01 CA259273.

## CONFLICT OF INTEREST STATEMENT

Scott A. Armstrong has been a consultant and/or shareholder for Neomorph, Hyku Therapeutics, C4 Therapeutics, Nimbus Therapeutics, Accent therapeutics and Stelexis therapeutics. Scott A. Armstrong has received research support from Janssen and Syndax. Scott A. Armstrong is an inventor on a patent related to MENIN inhibition WO/2017/132398A1. Michael W. M. Kühn receives honoraria and is a consultant for Pfizer, Kura Oncology, Jazz Pharmaceuticals, Bristol‐Myers Squibb/Celgene Abbvie, Servier, Johnson&Johnson, and Blueprint; is on the speakers bureau of Gilead and received travel support from Abbvie, Servier, Johnson&Johnson, Bristol‐Myers Squibb/Celgene, and Daiichi Sankyo. Florian Perner received travel support from Syndax Pharmaceuticals and CHARM Therapeutics. Jayant Y. Gadrey has no conflicts of interest to disclose.
